# Loss of Ribosomal Protein L11 Affects Zebrafish Embryonic Development through a p53-Dependent Apoptotic Response

**DOI:** 10.1371/journal.pone.0004152

**Published:** 2009-01-08

**Authors:** Anirban Chakraborty, Tamayo Uechi, Sayomi Higa, Hidetsugu Torihara, Naoya Kenmochi

**Affiliations:** Frontier Science Research Center, University of Miyazaki, Miyazaki, Japan; Baylor College of Medicine, United States of America

## Abstract

Ribosome is responsible for protein synthesis in all organisms and ribosomal proteins (RPs) play important roles in the formation of a functional ribosome. L11 was recently shown to regulate p53 activity through a direct binding with MDM2 and abrogating the MDM2-induced p53 degradation in response to ribosomal stress. However, the studies were performed in cell lines and the significance of this tumor suppressor function of L11 has yet to be explored in animal models. To investigate the effects of the deletion of L11 and its physiological relevance to p53 activity, we knocked down the *rpl11* gene in zebrafish and analyzed the p53 response. Contrary to the cell line-based results, our data indicate that an L11 deficiency in a model organism activates the p53 pathway. The L11-deficient embryos (morphants) displayed developmental abnormalities primarily in the brain, leading to embryonic lethality within 6–7 days post fertilization. Extensive apoptosis was observed in the head region of the morphants, thus correlating the morphological defects with apparent cell death. A decrease in total abundance of genes involved in neural patterning of the brain was observed in the morphants, suggesting a reduction in neural progenitor cells. Upregulation of the genes involved in the p53 pathway were observed in the morphants. Simultaneous knockdown of the *p53* gene rescued the developmental defects and apoptosis in the morphants. These results suggest that ribosomal dysfunction due to the loss of L11 activates a p53-dependent checkpoint response to prevent improper embryonic development.

## Introduction

The ribosome is an essential complex macromolecule responsible for protein synthesis in all organisms. In eukaryotes, a mature ribosome consists of 79 RPs and four rRNA species [1], [2]. RPs are important in the formation of a fully functional ribosome because they facilitate specific rRNA conformational changes during the assembly of the ribosomal subunits [3]. Besides their role in ribosome biogenesis, many RPs are believed to have important functions in various other cellular processes [4], [5]. Although recent high resolution crystallography results have demonstrated the structure of various RPs and their interaction with rRNAs in the ribosome [6], [7], such specific functions of RPs have yet to be investigated.

Haploinsufficiency of RPs in *Drosophila* leads to the *Minute* mutants, relatively normal flies with short thin bristles and delayed development [8], [9]. Such phenotypes are believed to be due to a reduced protein synthesis capacity of their ribosomes. However, recent reports indicate that mutations in RPs in vertebrates lead to specific phenotypes that are unlike the nonspecific general phenotypes typically expected for housekeeping genes. For example, a mutation in *Rpl24* in mice results in the ‘Belly spot and tail’ (*Bst*) mutant, which displays a kinked tail, a white ventral midline spot, and other skeletal deformities [10]. In zebrafish, malignant peripheral nerve sheath tumors (MPNSTs) are associated with haploinsufficiency of 11 RP genes, suggesting a role for RPs in tumorigenesis [11]. RP mutations have been observed in human diseases as well. Four RP genes, *RPS19 RPS24*, *RPS17*, and *RPL35a* [12]–[15] are known to be mutated in Diamond-Blackfan anemia (DBA; MIM 105650), a congenital erthyroid aplasia characterized by macrocytic anemia. *RPS14* has been implicated as the responsible gene in 5q^−^ syndrome, a disease that leads to a severe decrease in erythrocyte numbers in the bone marrow, resulting in a macrocytic anemia similar to DBA [16]. Our previous systematic knockdown analyses of RP genes in zebrafish produced gene-specific phenotypes that depended on the RP gene targeted [17], [18]. Thus, many RPs may have distinct functions, besides their involvement in ribosome formation, which are currently unknown. Investigating these unknown functions of RPs using animal models is vital for understanding the role of ribosomal proteins in human diseases.

L11 is one such RP proposed to have a dual physiological role; it is thought to participate in ribosomal assembly and in regulation of p53 activity [19]. Studies in human cell lines have demonstrated that L11 binds to MDM2, prevents MDM2-mediated p53 ubiquitination and degradation, stabilizes and activates p53, and induces cell cycle arrest [19], [20]. Overexpression of L11 or actinomycin D-induced ribosomal stress increases this L11-MDM2 interaction and subsequent p53 stabilization [21].

The p53 protein plays a critical role in the suppression of tumorigenesis; loss of p53 function is associated with the majority of human cancers. Activation of p53 induces cell cycle arrest, apoptosis, or DNA repair when cells are damaged or stressed [22]. Because of its inhibitory effects on cell growth, p53 is maintained at a low steady-state level in normal conditions by its negative regulator, MDM2, through ubiquitin-dependent p53 degradation [23]. Recent findings indicate that alterations in ribosome biogenesis are linked to the p53 pathway. Various studies have demonstrated that mutations in nucleolar proteins involved in rRNA synthesis or ribosomal subunit assembly affect ribosome synthesis and activate a p53 response through a nucleolus mediated stress [24]–[26]. Nucleolus is the major site of ribosome production [27] and it has been shown that disruption of the nucleolus is required for p53 activation and stabilization [28].

We report here that a deficiency in L11 activates the p53 pathway, and absence of L11 disrupts the normal embryonic development of zebrafish, presumably through a p53-mediated apoptotic response.

## Results

### Knockdown of *rpl11* leads to morphological defects mainly in the developing brain

**Figure 1:**
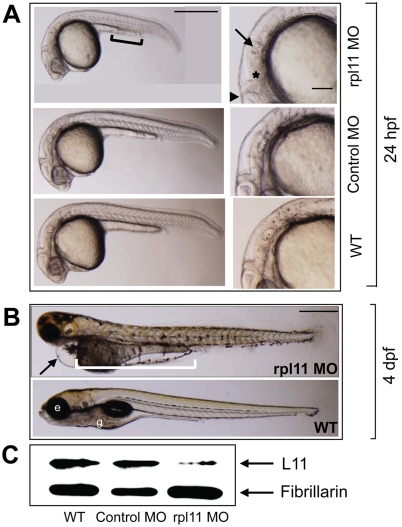
L11 deficiency causes morphological defects in zebrafish. (A) Lateral views of morphants (rpl11 MO, upper panel), controls (control MO, middle panel), and wild-type embryos (WT, lower panel) at 24 hpf. The morphants are smaller in size to the control embryos and the yolk sac extension is incomplete (black solid line). The right hand panel shows a close-up of the head region. The morphants display deformities in various parts of the brain, such as the midbrain-hindbrain boundary (filled black triangle), hindbrain (asterisk), and otolith (arrow). (B) Morphological observation of morphants and wild-type embryos at 4 dpf. The morphants have a smaller head and eyes, severe edema of the heart (arrow, upper panel), and incomplete yolk consumption with no gut (white solid line, upper panel) compared to wild-type embryos. (C) Western blot analysis of L11 in the morphants and control embryos with fibrillarin as a loading control. All images are anterior to the left. Scale bars: *A*, 500 µm (left panel), 100 µm (right panel); *B*, 500 µm. e, eye; g, gut.

Zebrafish L11 shares 95% amino acid identity with its human ortholog. We targeted this gene using an Morpholino antisense oligo (MO) aimed at its translation initiation site. Based on our previous data [17], we injected rpl11 MO and control MO (a five base mismatch of rpl11 MO) at a predetermined concentration (50 pg/embryo) into one-cell zebrafish embryos and analyzed the resulting phenotypes. At 24 hours post-fertilization (hpf), the morphants displayed an overall reduction of body length with an uncharacteristic cloudiness or opacity in the head region. However, the trunk region appeared to be relatively normal (Figure 1A, left panel). We also observed other phenotypes commonly associated with RP morphants [17], such as hypoplasia of the yolk sac extension, abnormalities in the ear, and delayed pigmentation of the eyes. Close up examination of the brain revealed improper differentiation, such as an enlarged forebrain, indistinct isthmic constriction at the midbrain-hindbrain boundary (mhb), and a malformed hindbrain (Figure 1A, right panel). The embryos injected with the control MO did not display any such mutant phenotypes (Figure 1A, middle row). By 4 days post-fertilization (dpf), the head and the eyes were significantly smaller in the morphants and the heart displayed pericardial edema. Improper yolk consumption and absence of a proper gut was also evident (Figure 1B). All the morphants died by 6–7 dpf. A reduction in the L11 protein level was confirmed using a rabbit polyclonal antibody specific to human L11 [19]. At 24 hpf, L11 was dramatically decreased in the rpl11 MO-injected embryos, whereas injection of control MO did not alter L11 levels (Figure 1C).

### rpl11 deficiency associated morphological defects are rescued by coinjection of *rpl11* mRNA

**Figure 2:**
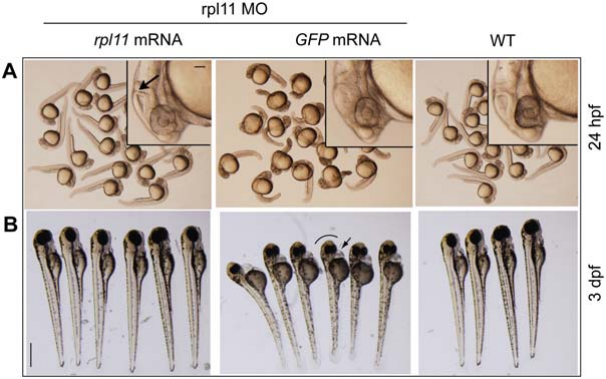
Morphological defects in L11-deficient embryos are rescued by *rpl11* mRNA. (A) Morphology of L11-deficient embryos (24 hpf) coinjected separately with *rpl11* mRNA and *GFP* mRNA. *rpl11* mRNA (25 ng/µl) coinjected with rpl11 MO (0.5 µg/µl) completely recovers the body length in 24 hpf embryos, but *GFP* mRNA does not produce any recovery. Insets show the rescue of brain parts such as midbrain-hindbrain boundary (arrow) in the embryos coinjected with rp*l11*mRNA, but not in *GFP* mRNA–coinjected embryos. (B) Morphological observation of the *rpl11* mRNA and *GFP* mRNA–coinjected embryos at 3 dpf. The *rpl11* mRNA–coinjected embryos have proper head and eye phenotypes, similar to those of the wild-type embryos, whereas *GFP* mRNA-coinjected embryos display smaller heads and eyes (black curved line) and pericardial edema (arrow). Scale bars: A, inset, 100 µm; B, 500 µm.

Coinjection of MO-resistant *rpl11* mRNA at 25 ng/µl (100 pl/embryo), but not overexpression of *GFP* mRNA, rescued the developmental defects in the morphants. This indicates that the altered phenotypes in the morphants were specifically due to a loss of L11. When observed at 24 hpf, the head region and the overall body length were completely recovered in *rpl11* mRNA-coinjected embryos, whereas *GFP* mRNA-coinjected embryos still displayed morphant phenotypes (Figure 2). Various parts of the brain, such as the isthmus of the midbrain-hindbrain boundary, the forebrain, and the hind brain, were rescued in *rpl11* mRNA-coinjected embryos (Figure 2, insets in the upper panel). By 3 dpf, the rescue of the morphants was more evident (Figure 2, lower panel).

### Neuronal cell abundance, but not fate, is affected by rpl11 deficiency in the brain

**Figure 3:**
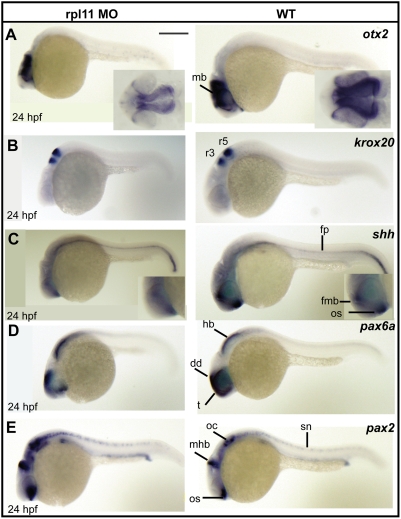
The L11-deficient embryos exhibit reduced abundance of neuronal progenitor cells and aberrant expression of transcription factors. (A) Expression of *otx2* at 24 hpf. The morphants show a reduction in total abundance of *otx2*, but its pattern of expression is same as that of wild-type embryos. The inset shows the dorsal view of the midbrain region. (B) *krox20* expression at the hindbrain in 24 hpf embryos. The morphants show a proper hindbrain patterning compared to the wild-type embryos. (C) Expression of s*hh* in morphants and wild-type embryos at 24 hpf. The morphants show a marginal reduction in the forebrain midbrain boundary (fmb, inset in C), but floor plate (fp) expression is unaffected. (D) Expression of *pax6a* in morphants and wild-type embryos at 24 hpf. The morphants show a reduction in total abundance of *pax6a*, but its pattern of expression is same as that of wild-type embryos (E) The morphants show an increased level of *pax2* expression compared to wild-type embryos. The midbrain hindbrain boundary (mhb) expression of *pax2* is short and wide in the morphants. *pax2* expression at the optic stalk and the otic capsules is also increased in the morphants. All images are in lateral views with anterior to the left. Scale bar: 250 µm. hb, hindbrain; dd; dorsal diencephalon; t, telencephalon; r3, rhombomere 3; r5, rhombomere 5; fmb, forebrain-midbrain boundary; os, optic stalk; mhb, midbrain-hindbrain boundary; oc, otic capsule; sn, spinal chord neurons.

Since the morphants displayed altered phenotypes mainly in the brain region during early developmental stages (24 hpf), we performed *in situ* hybridization to analyze the expression pattern of several marker genes involved in neural patterning of the brain. The expression of *otx2*, a mid brain marker, showed a decrease in total abundance in the morphants, compared to the wild-type embryos (Figure 3A). The expression of *krox20*, a marker specific for hindbrain segmentation, was unaffected in the morphants (Figure 3B), although they displayed a morphologically deformed hindbrain. We also analyzed the expression pattern of *shh*, a neuronal marker specific for the floor plate organizer (fp) and the forebrain/midbrain boundary (fmb). The expression of *shh* at fp was unaffected, but the expression level at fmb was marginally decreased in the morphants (Figure 3C, inset), indicating that decreased axis length in the morphants is not due to the loss of floor plate formation. However, the localization of these neural markers in the morphants was same as that of wild-type embryos, suggesting that cell fate was unaffected in the morphants. Thus, it appears that L11 deficiency, although results in neural cell death, does not change the patterning and localization of the neural cells in the brain.

### rpl11 deficiency leads to misregulated expression of transcription factors

We investigated whether the defects in the brain could be due to an aberrant expression of transcription factors that play important role in vertebrate morphogenesis, particularly that of the brain. *Pax2* and *pax6a*, members of the paired box family of transcription factors are important for the brain development in zebrafish. *In situ* analysis of *pax6a* revealed a decrease in the total abundance (reduction in staining intensity) in the forebrain region of the morphants (Figure 3D). However, an overexpression of *pax2* transcript was observed in the morphants at 24 hpf (Figure 3E).

Thus, it appears that the *pax6a* and *pax2* signaling pathway is affected by L11 deficiency, although the exact mechanism of such an effect remains to be defined. Overexpression of *pax2* has been observed in zebrafish *aussicht* (*aus*) mutant, a dominant mutant identified by ENU screening, which exhibit defects in the differentiation of forebrain, midbrain and eyes [29]. This corroborates our data that the specific defects in the brain development, as seen in L11-deficient embryos, may lead to aberrant expression of these markers. The analysis of the expression pattern of these neural markers in control MO-injected embryos revealed similar levels to those in wild-type embryos (Figure S1), confirming that the decrease in abundance of these neural marker genes were specific to *l11* deficiency.

### Developmental defects in L11-deficient embryos are due to increased cellular apoptosis

**Figure 4:**
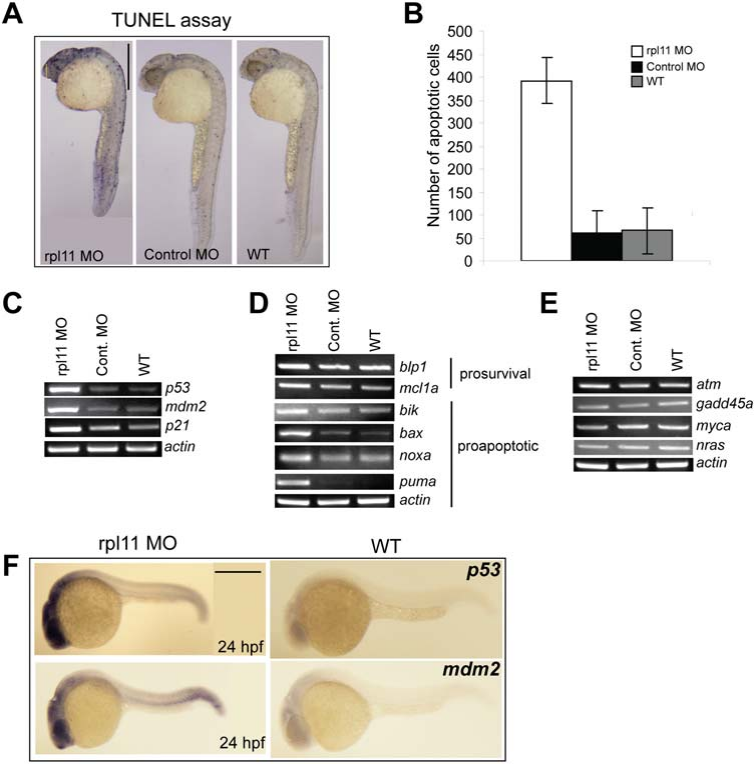
L11 deficiency results in increased cellular apoptosis mediated by p53. (A) TUNEL staining at 24 hpf. The morphants show massive apoptosis (blue dots indicate the TUNEL-positive cells) concentrated primarily in the head region compared to a few scattered positive cells in control and wild-type embryos. (B) Histogram showing the number of apoptotic cells in the head region. Values represent the mean of total number of apoptotic cells counted in the head region of three embryos each for the morphant, control, and wild-type embryos. (C-E) Semi-quantitative RT-PCR of *p53*, p53-target genes, *Bcl-2* family genes, *atm*, *gadd45a,* m*yca*, and *nras* transcript levels relative to *actin* in the morphant, control, and wild-type embryos at 24 hpf. (F) *In situ* analysis of *p53* and *mdm2* at 24 hpf. The morphants show increased expression of *p53*, exclusively in the head region. *mdm2* expression, although more ubiquitous, is also increased in the morphants, particularly in the head region. All images are in lateral views with anterior to the top in A and left in F. Scale bars: A, 500 µm.

To determine whether the developmental defects observed in the morphants were due to apoptosis, we analyzed the morphants and the control embryos using a TUNEL assay. Extensive apoptosis, as indicated by increased numbers of TUNEL-positive cells, were observed in the morphants at 24 hpf (Figure 4A and 4B). In contrast, the control MO-injected embryos and wild-type embryos displayed only a few scattered apoptotic cells, at levels consistent with previous observations [30]. Moreover, TUNEL-positive cells in the morphants were concentrated in the head regions (Figure 4A) that displayed morphological abnormalities (Figure 1A, right panel).

### p53 pathway is activated in L11-deficient embryos

To examine whether a p53-pathway was activated with the loss of L11, we analyzed the expression of *p53* and its target genes in L11-deficient embryos. Semi-quantitative RT-PCR at 24 hpf showed marked upregulation of *p53* mRNA in the morphants (Figure 4C). Upregulation of *p21*, an important kinase and a transcriptional target of p53, and *mdm2*, the negative regulator and downstream target of p53, was also seen in L11-deficient embryos but not in control MO-injected and wild type embryos (Figure 4C). The proapoptotic Bcl-2 family genes (*bik, bax, noxa,* and *puma*), which are regulated by p53, showed transcriptional upregulation in the morphants compared to the control embryos (Figure 4D), whereas the prosurvival markers (*blp1*, *mcl1a*) were not altered in the morphants, indicating an activated apoptotic pathway; this result was consistent with the TUNEL data (Figure 4A). In order to confirm that the increase in p53 activity was specifically due to *rpl11* knockdown, we analyzed the transcript levels of the kinase *atm*, which is activated during DNA damage; *gadd45a*, a p53 target gene activated during DNA damage; and the oncogenes *myca* and *nras*. There was no difference in the transcript levels of these genes between the morphants and the control embryos (Figure 4E), suggesting that DNA damage or oncogene overexpression is not responsible for p53 activation in L11-deficient embryos.

To determine whether p53 activation was an immediate effect of L11 loss-of-function, we performed a time course analysis of *p53* and its target genes in L11-deficient and wild-type embryos at various stages post fertilization (4, 8, 12, and 48 hpf). Our data indicate that activation of *p53* and its target genes in L11-deficient embryos coincided with the start of zygotic transcription during embryogenesis (Figure S2). Zebrafish embryos do not begin zygotic transcription until 3 hpf and early development proceeds in the absence of RNA synthesis. At 4 hpf, a stage immediately after the start of zygotic transcription, there was no difference in the transcript levels of *p53* and its target genes between L11-deficient and wild type embryos (Figure S2). However, by 8 hpf, marginal upregulation of *p53* and *p21* transcripts were evident in L11-deficient embryos. By 12 hpf, *mdm2*, *bax*, and *puma* also showed transcriptional upregulation in the morphants. At 48 hpf, *p53* and its target genes were significantly upregulated in the L11-deficient embryos (Figure S2).

Finally, to examine if the expression of *p53* and its target genes is localized specifically in the head region of the morphants, we performed *in situ* hybridization of *p53* and one of its target gene, *mdm2*. L11-deficient embryos showed increased *p53* expression, almost exclusively in the head region, as compared to the wild-type embryos (Figure 4F). Similarly, *mdm2* expression, although more ubiquitous than *p53*, was increased in the morphants particularly in the head region (Figure 4F). These data indicates that p53 activation in the brain was consistent with the neural cell death and increased apoptosis in the morphants.

### Suppression of p53 activity rescues the developmental defects and apoptosis in L11-deficient embryos

**Figure 5:**
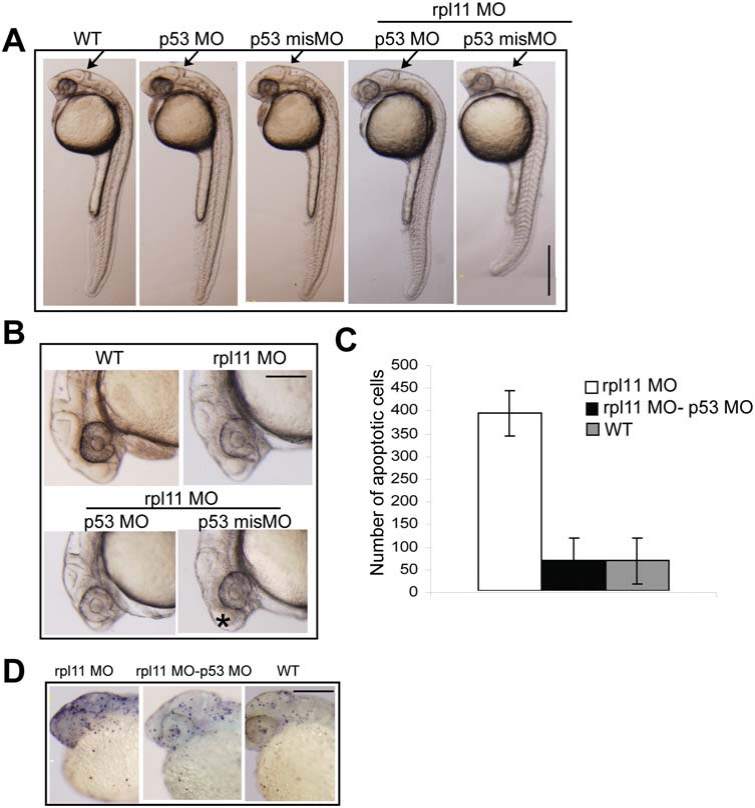
Inactivation of *p53* rescues the morphological defects and apoptosis in L11-deficient embryos. (A, B) Morphology of L11-deficient embryos coinjected with either p53 MO or p53 misMO compared to the control embryos at 24 hpf. The overall body length, midbrain-hindbrain boundary (arrow in A), and enlarged forebrain (asterisk in B) are rescued in p53 MO, but not in p53 misMO-injected L11-deficient embryos. (C, D) TUNEL staining of p53 MO injected L11-deficient embryos. The numbers of apoptotic cells (TUNEL-positive cells in D) are reduced, almost similar to those in wild-type embryos, in p53 MO-coinjected embryos compared to rpl11 MO alone–injected embryos. All images are in lateral views with the anterior to the top in A and D and to the left in B. Scale bars: A, 500 µm; B and D, 200 µm.

Since an L11 deficiency activates a p53-dependent apoptotic response, we examined whether simultaneous knockdown of *p53* and *rpl11* could rescue the developmental defects and apoptosis of L11-deficient embryos. A previous study has shown that 4 ng/embryo of p53 MO specifically blocks the synthesis of the zebrafish p53 [31]. To reduce the MO-induced stress to the embryos in a simultaneous knockdown, we used 50 pg/embryo of p53 MO, which was sufficient to perfectly suppress p53 activity (Figure S3). We coinjected rpl11 MO and p53 MO, or their respective control MOs, into zebrafish at the one-cell stage, and analyzed the resulting phenotypes. Coinjection of rpl11 MO and p53 MO could rescue the developmental defects, but p53 misMO-coinjected embryos displayed the same developmental defects as those in L11-deficient embryos (Figure 5A). The mhb and the enlarged forebrain were rescued in rpl11 MO/p53 MO-coinjected embryos but not in p53 misMO-coinjected embryos (Figure 5B).

To assess the number of apoptotic cells in the coinjected embryos, we performed a TUNEL assay. As expected, coinjection of rpl11 MO and p53 MO resulted in a substantial reduction of TUNEL-positive cells, to levels nearly the same as those in wild-type embryos (Figure 5C and D). Although the p53 MO-injected *rpl11* morphants survived longer than the embryos injected with only rpl11 MO, they eventually died between 9 and 10 dpf (data not shown). Thus, the loss of p53 function leads to suppression of early apoptosis and developmental defects in L11-deficient embryos, but fails to rescue the ribosomal dysfunction that is associated with L11 deficiency. These results indicate that a p53-mediated apoptotic response is activated upon *rpl11* knockdown in zebrafish.

## Discussion

In recent years, three RPs, L5, L11, and L23, have been shown to regulate p53 activity by binding to MDM2 specifically in response to a ribosomal stress [19], [32], [33] which has led to speculations about a novel ribosome-mediated p53 checkpoint mechanism. Of these three RPs, L11 has been the most extensively studied for its role in the MDM2-p53 pathway. Previous reports have shown that overexpression of L11 in ribosome-perturbed cells, but not in DNA-damaged or oncogene-activated cells, greatly increases L11-MDM2 binding in a p53-dependent manner both in normal and in tumor cells [19], [34]. Moreover, the direct binding of L11 (central domain, amino acids 51–108) is essential for MDM2 stabilization [35]. Taken together, these results indicate that L11 is a major inhibitor of the MDM2-p53 feedback loop in response to ribosome stress.

To understand the importance of this association in a model system like zebrafish, we reduced the level of L11 protein by a knockdown strategy and analyzed the phenotypes. The MO used in this study was designed to target the initiation codon of *rpl11* mRNA so that the synthesis of the entire protein was blocked. Since L11 is required for ribosome assembly, knocking down this protein should result in a ribosomal stress. And as the association of L11-MDM2 greatly increases in response to ribosomal stress, it is expected that removal of L11 in a system without DNA damage or oncogenic overexpression should result in reduced L11-MDM2 binding or increased MDM2-p53 binding.

Contrary to expectations, our data show that an L11 deficiency activates the p53 pathway leading to developmental abnormalities, apoptosis, and embryonic lethality. Interference with ribosomal biogenesis by actinomycin D further increased the level of apoptosis in L11-deficient embryos (data not shown), as ribosomal biogenesis was already compromised in these embryos.

We have clearly shown that this p53 activation and the associated neural cell death in rpl11 MO-injected embryos is not an off-target effect of morpholino [36] but a result of specific loss of L11 protein. First, our morphants were phenotypically similar (Figure S4) to a zebrafish mutant line having a viral insert in the *rpl11* gene (*rpl11^hi3820btg^*). This mutant was identified in a forward genetic screen for essential genes [37]. Second, we have verified the loss of L11 protein by using a human L11 antibody. Third, the phenotype of the L11 morphants was completely rescued by MO resistant *rpL11* mRNA (Figure 2). Fourth, upregulation of p53 response in other RP mutants [38] and ribosome-associated protein mutants [39] validates our data that p53 activation in L11-deficient embryos is not due to non-specific morpholino response but due to the specific loss of L11 protein. Finally, the control morpholino, which is a five base mismatch of the original MO, did not result in any phenotype, apoptosis or upregulation of the p53 pathway (Figure 1A, Figure 4).

It can be argued that L5 and L23, which also have MDM2 binding properties, may be responsible for the observed increased p53 activity in L11-deficient embryos. In the absence of proper ribosome assembly, these proteins may remain free in the nucleus and bind to MDM2, thereby activating p53. To exclude the possibility of an L5/L23-MDM2 association in L11-deficient embryos, we simultaneously knocked down the *rpl5*, *rpl11*, and *rpl23* genes in zebrafish and analyzed the phenotypes. Our results showed that even in the absence of these p53 regulatory RPs, the embryos displayed severe morphological deformities and a high level of apoptosis, indicating an activated p53 response (Figure S5).

Therefore, it is likely that improper ribosome biogenesis, rather than the loss of a specific function of L11, activates the p53 dependent response and leads to morphological defects and increased apoptosis in L11-deficient embryos. Deficiency of any RP, regardless of its role in p53 regulation, may lead to such a consequence. RPS19 deficiency in zebrafish was reported to result in transcriptional activation of *p53* [38]. Moreover, a number of zebrafish lines with mutations in *rps8*, *rps11*, and *rps18*, identified through a large scale insertional mutagenesis screen [37], showed strong upregulation of p53 as well as p53 target genes, including *p21* and *bax* [38]. *Rps19* and *Rps20* mutations in mice were reported to cause p53 activation and stabilization leading to pleiotrophic effects [40]. Haploinsufficiency of the RPs, S6 or L22, induce developmental defects and apoptosis in mice [41], [42] and both involve activation of the p53 checkpoint. In unpublished studies, we also found that knockdown of *rps24* and *rpl35* in zebrafish results in morphological abnormalities and increased apoptosis, indicating an upregulated p53 response. Thus, an activation of the p53 seems to be a common response to ribosomal stress.

Upregulation of a truncated isoform of p53, Δ113p53, which results from an alternative start site in the 4^th^ intron and lacks part of the DNA binding domain [43], was suggested to be a diagnostic signature of off-target neural death by MOs [36]. Although we found upregulation of Δ113*p53* transcript in L11-deficient embryos (data not shown), it was consistent with the data from other RP mutants [38] and ribosome-associated protein mutants in zebrafish [39] which reported upregulation of Δ113p53 isoform.

How does a ribosome biogenesis stress activate the p53 pathway? Previous studies have suggested that mutations in nucleolar proteins involved in ribosome assembly result in ribosomal stress and nucleolus disruption resulting in the release of these three RPs (L5, L11, and L23). These free RPs then bind to MDM2 and activate p53 [44], [45]. However, our data indicates that even in the absence of these RPs, p53 still gets activated. We assume that in case of an RP (L11) deficiency, ribosomal subunit assembly will be severely affected and several nucleolar proteins that would otherwise participate in the assembly may remain unutilized. Accumulation of these unused processing factors within the nucleolus may be sensed as a stress signal leading to an activated p53 response. Interestingly, we observed upregulation of *bop1*, *rrs1*, and *pes*, nucleolar proteins required for the nuclear assembly and export of the 60S pre-ribosomal subunit [24]–[26], [46] in L11-deficient embryos (data not shown).

However, it still remains unclear why an L11 deficiency results in restricted phenotypes, mainly in the developing brain, when ribosomes are so critical and essential for all cells. It has been suggested that in zebrafish, initial embryonic development relies on a large pool of maternal ribosomes, and that the brain may be the first tissue to “run out” of maternal ribosomes [26]. The brain belongs to the ‘highly proliferative tissue zone’ during early zebrafish development. During somitogenesis, the rate of ribosome production may vary between organs and, because of its large size and rapid development, this rate may be highest in the brain. At 24 hpf, the expression pattern of many assembly factors and processing proteins, such as *bop1*, *rrs1*, and *bap28*, show a spatial distribution, with the highest concentration in the brain region [26], [47], supporting the notion that ribosome production is highest in the brain. Therefore, absence of L11 leads to impaired ribosome synthesis, which results in phenotypes specific to the brain region.

As mentioned previously, a number of cellular studies have shown RPs (L5, L11 and L23) as negative regulator of p53 because of their MDM2 binding property. A decrease of these RPs should allow an increase of free MDM2, thereby decreasing the levels of p53. However, our results indicate that in zebrafish loss of L11 individually or in combination with other two RPs (L5 and L23), actually increases the p53 activity, rather than decreasing it. Recently, it has been shown that RP mutant zebrafish which develop MPNSTs [11], fail to synthesize p53 protein, but this observed loss of p53 is not due to the L5/L11/L23-MDM2 regulation [48]. Therefore, it seems likely that some other mechanism of p53 activation exists in the cell which links RPs to ribosomal biogenesis stress. We speculate that in a model system like zebrafish, loss of L11 would affect its primary function, namely participation in ribosome assembly. Accumulation of defective ribosomes due to RP deficiency would be sensed as a stress signal, presumably a nucleolar stress, leading to programmed cell death. A failure to activate this p53-dependent checkpoint could potentially lead to uncontrolled cell growth, as seen in tumorigenesis. Such a consequence may be true for other RPs, regardless of their MDM2-binding properties.

Future studies need to be directed towards understanding the underlying mechanism of how RP insufficiency activates the p53 pathway and what roles individual RPs play in this pathway. Such studies could provide valuable insights into the relationship between ribosomal abnormalities and diseases.

## Materials and Methods

### Morpholino oligonucleotide injections

Morpholinos were obtained from Gene Tools, LLC (USA). For *rpl11*, sequences within 25 bases around the translation start site (AUG) were used to design the start codon–targeted *rpl11* Morpholino (rpl11 MO). For control, an rpl11 mismatch Morpholino (control MO) that included five mispaired bases was used. The Morpholino sequences are displayed in Table S1. The sequences of p53 MO and p53 misMO were described by Langheinrich *et al* [31]. Based on our previous injection experiments [17], rpl11 MO or p53 MO at 0.5 µg/µl (∼50 pg/embryo) were injected into the blastomere of one-cell stage embryos using an IM-30 Electric Microinjector (Narishige, Japan). Control MOs were injected at the same volume as those used for the respective Morpholinos.

### Western blot analysis

Embryos (24 hpf) were completely deyolked in Ginzburg Fish Ringer buffer (55mM NaCl, 1.8mM KCl, 1.25mM NaHCO_3_) and washed with PBS. The embryos were collected by centrifugation and lysed with 2x Laemmli's buffer for 30 min followed by centrifugation to remove the debris. After separation in a 12.5% SDS-PAGE, the proteins were blotted onto a nitrocellulose filter, hybridized with rabbit polyclonal anti-human L11 antibody (1∶1000), and immunodetected using ECL plus Western Blot Detection System (GE Healthcare, UK).

### TUNEL assay

For detection of apoptotic cells in whole embryos, a TUNEL assay was performed using an *In Situ* Cell Death Detection kit-AP (Roche, Germany) according to a previously described protocol [49].

### Whole mount *in situ* hybridization (WISH)

Clones of the *in situ* markers were obtained from various labs (mentioned in the Acknowledgement section) and digoxigenin-labeled antisense RNA probes were generated with T7 or T3 RNA polymerases (Roche) according to the orientation of cDNA inserts. WISH was performed using the Thisse protocol [50] with the following modifications: 1) After the proteinase K digestion, the embryos were soaked in 2 mg/ml glycine-HCl/PBS for 5 min at room temperature followed by a 4% paraformaldehyde fixation, 2) Prehybridization and hybridization steps were carried out at 65°C, 3) tRNA was added at a concentration of 5 mg/ml to the hybridization buffer, and 4) The hybridization buffer used in washing steps contained tRNA and heparin.

### *In vitro* transcription of mRNA

To prepare the MO-resistant *rpl11* mRNA, four base changes were inserted in the Morpholino binding region of *rpl11* without changing the amino acids. The full length cDNA was PCR amplified with forward and reverse primers containing *Bam*H1 and *Eco*RI sites, respectively. The double-digested purified PCR product was then ligated to pCS2+ plasmid (kindly provided by Dr. K. Inoue, Kobe University, Japan) and capped *rpl11* mRNA was generated from 1 µg of *Not*1-linearized pCS2+/*rpl11* by SP6 RNA polymerase using the mMESSAGE mMACHINE kit (Ambion, USA). The synthesized RNA was purified by MEGAclear (Ambion). For *GFP* mRNA, the full length *GFP* cDNA inserted clone was digested with *Not*I followed by *in vitro* transcription as mentioned above.

### Semi-quantitative RT-PCR

Total RNA was extracted from 25–30 embryos using TRIzol Reagent (Invitrogen, USA) according to manufacturer's instructions. One microgram of total RNA was used as a template in a 20 µl RT-PCR reaction mixture using a one step RT-PCR kit (Qiagen, Germany). The RT-PCR conditions are as described by Berghmans *et al*. [49] except for a change in annealing temperature, which depended on the *Tm* value of the primers. The primers used in this study are listed in Table S2.

## Supporting Information

Table S1 The Sequences of Morpholinos Used in This Study
(0.03 MB DOC)

Table S2 List of RT-PCR Primers Used in This Study
(0.04 MB DOC)

Figure S1 Whole mount in situ hybridization of neural markers in control MO-injected and wild type embryos.The expression level and pattern of krox20, shh, and pax6a in control MO- injected embryos is exactly similar to wild-type embryos at 24 hpf. All images are in lateral views with anterior to the left. Scale bar: 250 µm
(6.35 MB TIF)

Figure S2 Time-course expression analysis of p53 and its target genes. Semi-quantitative RT-PCR of p53, p53 target genes (p21, mdm2) and apoptotic markers (bax and puma) transcript levels relative to actin in the morphants and wild-type embryos at 4, 8, 12, and 48 hpf.
(0.58 MB TIF)

Figure S3 Inhibition of p53 activity and suppression of apoptosis by p53 MO. Zebrafish embryos at the one-cell stage were injected with p53 MO and p53 misMO at 50 pg/embryo. At 24 hpf, the embryos were exposed to 400 mJ/cm UV light for 5 s. Six hours later, they were fixed for TUNEL staining and RT-PCR. (A) Semiquantitative RT-PCR of p53-target genes in UV-exposed embryos injected with p53 MO (50 pg/embryo) or p53 misMO (50 pg/embryo). The p53 response genes are completely downregulated in p53 MO-injected, but not in p53 misMO-injected, embryos. (B–E) TUNEL staining of p53 MO- and p53 misMO-injected embryos before and after UV exposure. The embryos injected with p53 MO show a complete inhibition of apoptosis (indicated by an absence of TUNEL-positive cells) after UV exposure, whereas p53 misMO-injected embryos show extensive apoptosis. All images are in lateral view with anterior to the left. Scale bar: 500 µm.
(3.42 MB TIF)

Figure S4 Phenotype of L11morphants at 2 day post fertilization (dpf). Morphology of L11 morphants (upper panel) shows slightly smaller head and eyes, round grey yolk with thin extension, identical to that observed in L11 mutant (rpl11hi3820btg). Lower panel is the wild-type embryo at day 2 dpf. All images are in lateral views with anterior to the left. Scale bar: 1mm
(7.92 MB TIF)

Figure S5 Morphological observation and TUNEL staining of embryos injected simultaneously with rpl5, rpl11, and rpl23 MOs.(A) Lateral views of 24 hpf embryos simultaneously injected with the three MOs. Severe morphological deformities with almost a complete absence of brain subdivisions (black arc) were observed in the morphants. (B) Dorsal and lateral views of the head region of 24 hpf embryos showing extensive apoptosis (blue dots). Scale bars: A, 250 µm; B, 100 µm.
(8.39 MB TIF)
