# Supplementary material for: Loss of Ribosomal Protein L11 Affects Zebrafish Embryonic Development through a p53-Dependent Apoptotic Response
Source: PLoS One. 2009 Jan 8;4(1):e4152. doi: 10.1371/journal.pone.0004152 (PMC2612748; doi:10.1371/journal.pone.0004152)
Supplement: Table S1 — The Sequences of Morpholinos Used in This Study (0.03 MB DOC) [file pone.0004152.s001.doc]

**Supplementary Tables**

**Table S1 The Sequences of Morpholinos Used in This Study**

| MO | Sequence |
| --- | --- |
| rpl11 MO | 5`-CTTCTTCTCGCTCTGGTCCGCCATG-3` |
| rpl11 misMO | 5`-CTTgTTgTCGCTgTGcTCCGCgATG-3` |
| rpl5 MO | 5`-ACCCATTTTGTGATCGTTTGTTCTC-3` |
| rpl23 MO | 5`**-**ACCACGTCCTCTCTTAGACATAGTC-3` |

The sequence complimentary to the initiation codon is underlined. Mispaired bases

are indicated in lower cases.
