# Supplementary material for: Loss of Ribosomal Protein L11 Affects Zebrafish Embryonic Development through a p53-Dependent Apoptotic Response
Source: PLoS One. 2009 Jan 8;4(1):e4152. doi: 10.1371/journal.pone.0004152 (PMC2612748; doi:10.1371/journal.pone.0004152)
Supplement: Table S2 — List of RT-PCR Primers Used in This Study (0.04 MB DOC) [file pone.0004152.s002.doc]

| Gene | Primer sequences |
| --- | --- |
| *tp53* (NM_131327) | Forward: 5'- ACCACTGGGACCAAACGTAG-3'  Reverse: 5'- CAGAGTCGCTTCTTCCTTCG-3' |
| *p21* (NM_001002717) | Forward: 5'-CCAGTCAGAATGAGCAGCAC -3'  Reverse: 5'- CAGGTGCTGGAATAGGATCG-3' |
| *gadd45a* (NM_001002216) | Forward: 5'- GGAGATAACGCAACGGAAAG-3'  Reverse: 5'- TTGCTTCTGTTCATCGTTCG-3' |
| *mdm2* (NM_131364) | Forward: 5'- GGCTTCCAGAAACACACTCC-3'  Reverse: 5'- ACTGACTGAATGGGCTCTCG-3' |
| *blp1* (AF317837) | Forward: 5'- CAGAGGAACTACCCCTGCAA-3'  Reverse: 5'- CATCCTCCTTGGCTTTGGAT-3' |
| *mcl1a* (NM_131599) | Forward: 5'- ACTCCGACGCTAAAGACGTG-3'  Reverse: 5'- GCCAGCTCTTGTTTTTGAGG -3' |
| *bik* (DQ860154) | Forward: 5'- CTGGACCTGCTGAGGTTGA-3'  Reverse: 5'- TGTAGTGCTGCGAGACCAGT-3' |
| *bax* (NM_131562) | Forward: 5'- GTGTGACCCCAGCCATAAAC-3'  Reverse: 5'- TCACCCTCTGTTCACCGTCT-3' |
| *puma* (DQ 860151) | Forward: 5'- GGAAAGCAGAGTGGACGAAC -3'  Reverse: 5'- GTCCCCGATTGTCCTCAGTT -3' |
| *noxa* (DQ 860152) | Forward: 5'- ATGGCGAAGAAAGAGCAAAC-3'  Reverse: 5'- CGCTTCCCCTCCATTTGTAT-3' |
| *β-actin* (NM_131031) | Forward: 5'- GCCCATCTATGAGGGTTACG-3'  Reverse: 5'- GCAAGATTCCATACCCAGGA-3' |
| *atm* (AJ605775) | Forward: 5`- AACGTCACTTTCACCTGGCCGG-3`  Reverse: 5`- GTCAATGTGGACCAGTTCTGAGGT-3` |
| *myca* (NM_131412) | Forward: 5`- AACAATTCTGGAACGGCATT-3`  Reverse: 5`- TAGTTGTGCTGGTGGGTGGA-3` |
| *nRas* (NM_131145) | Forward: 5`-CCCTCAGGACTGTAAGATGACTG-3`  Reverse:  5`-CGGTCACATGACTTCACAGG-3` |
| *rrs1* (BC055615) | Forward: 5`-AGAACCAAAATGGCTTCGTG-3`  Reverse: 5`-TCATAAGTCCACCACACTGCTT-3` |
| *pes* (NM_131030) | Forward: 5`-ATCCTCATGAGCCCAGACAC -3`  Reverse: 5`-CCCTTCAGTAGGGAAGTGGTC-3` |

**Table S2** List of RT-PCR Primers Used in This Study
