# Supplementary figures and images for: Loss of Ribosomal Protein L11 Affects Zebrafish Embryonic Development through a p53-Dependent Apoptotic Response
Source: PLoS One. 2009 Jan 8;4(1):e4152. doi: 10.1371/journal.pone.0004152 (PMC2612748; doi:10.1371/journal.pone.0004152)

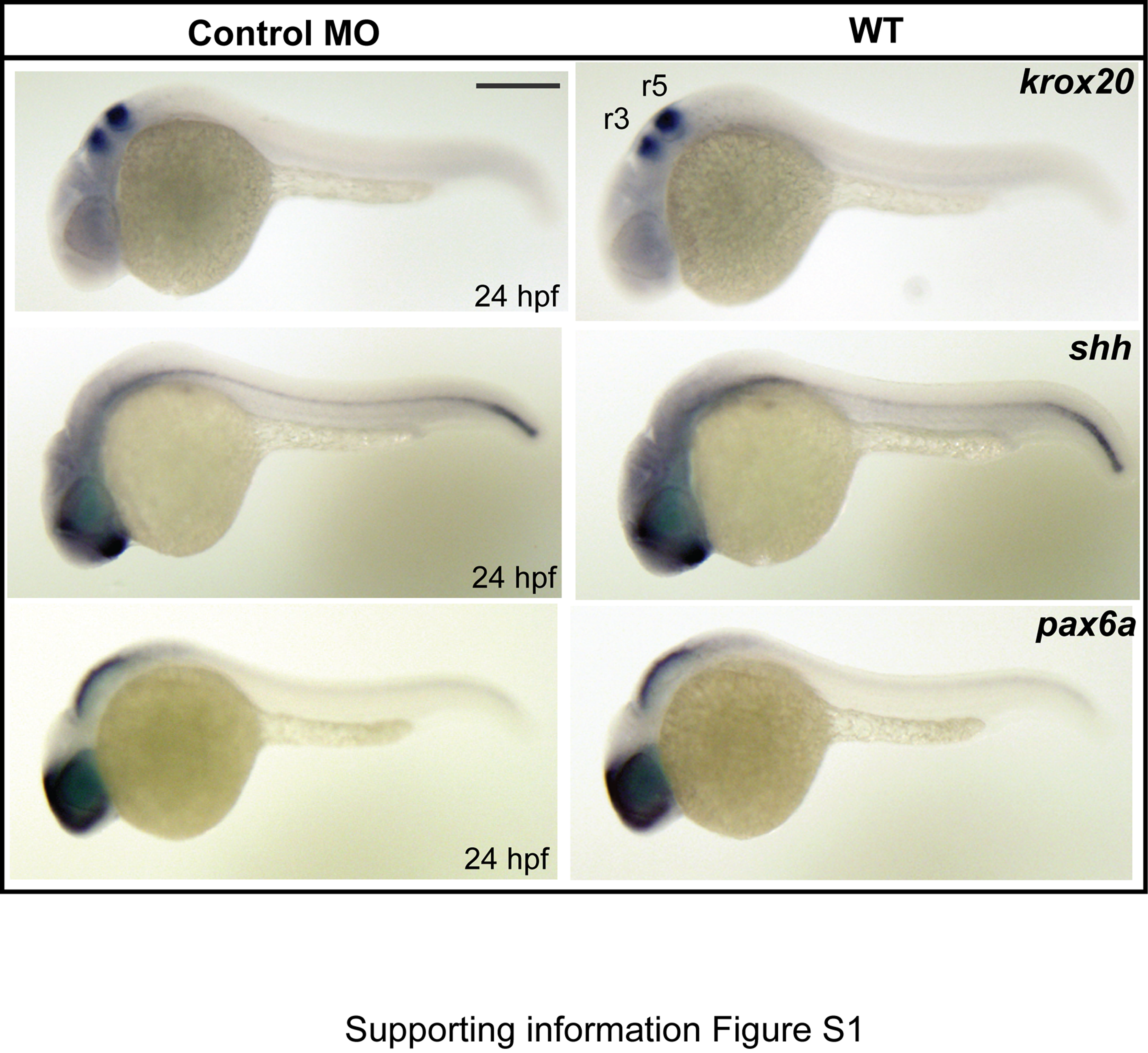

Supplement: Figure S1 — Whole mount in situ hybridization of neural markers in control MO-injected and wild type embryos.The expression level and pattern of krox20, shh, and pax6a in control MO- injected embryos is exactly similar to wild-type embryos at 24 hpf. All images are in lateral views with anterior to the left. Scale bar: 250 µm (6.35 MB TIF) [file pone.0004152.s003.tif]

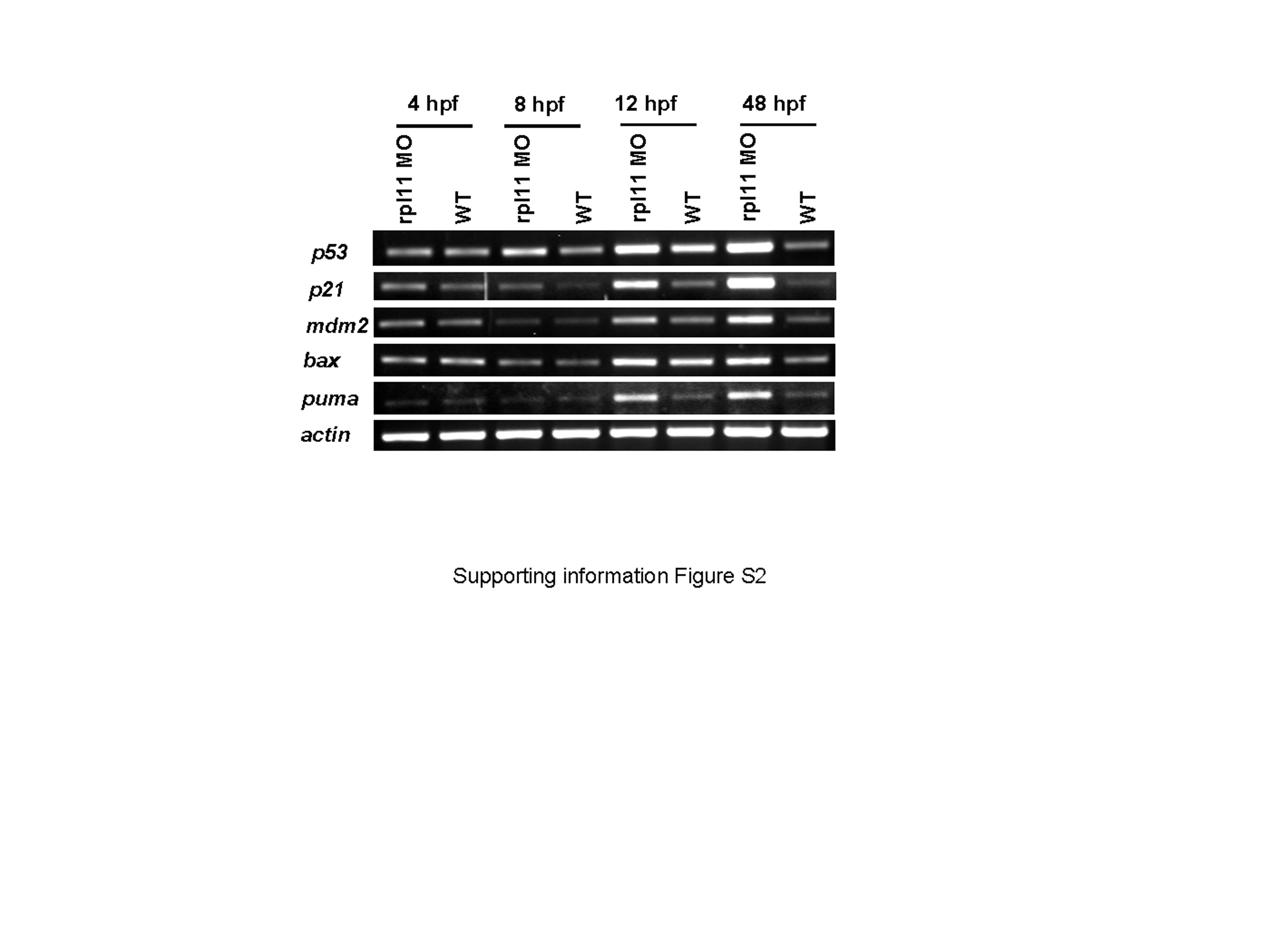

Supplement: Figure S2 — Time-course expression analysis of p53 and its target genes. Semi-quantitative RT-PCR of p53, p53 target genes (p21, mdm2) and apoptotic markers (bax and puma) transcript levels relative to actin in the morphants and wild-type embryos at 4, 8, 12, and 48 hpf. (0.58 MB TIF) [file pone.0004152.s004.tif]

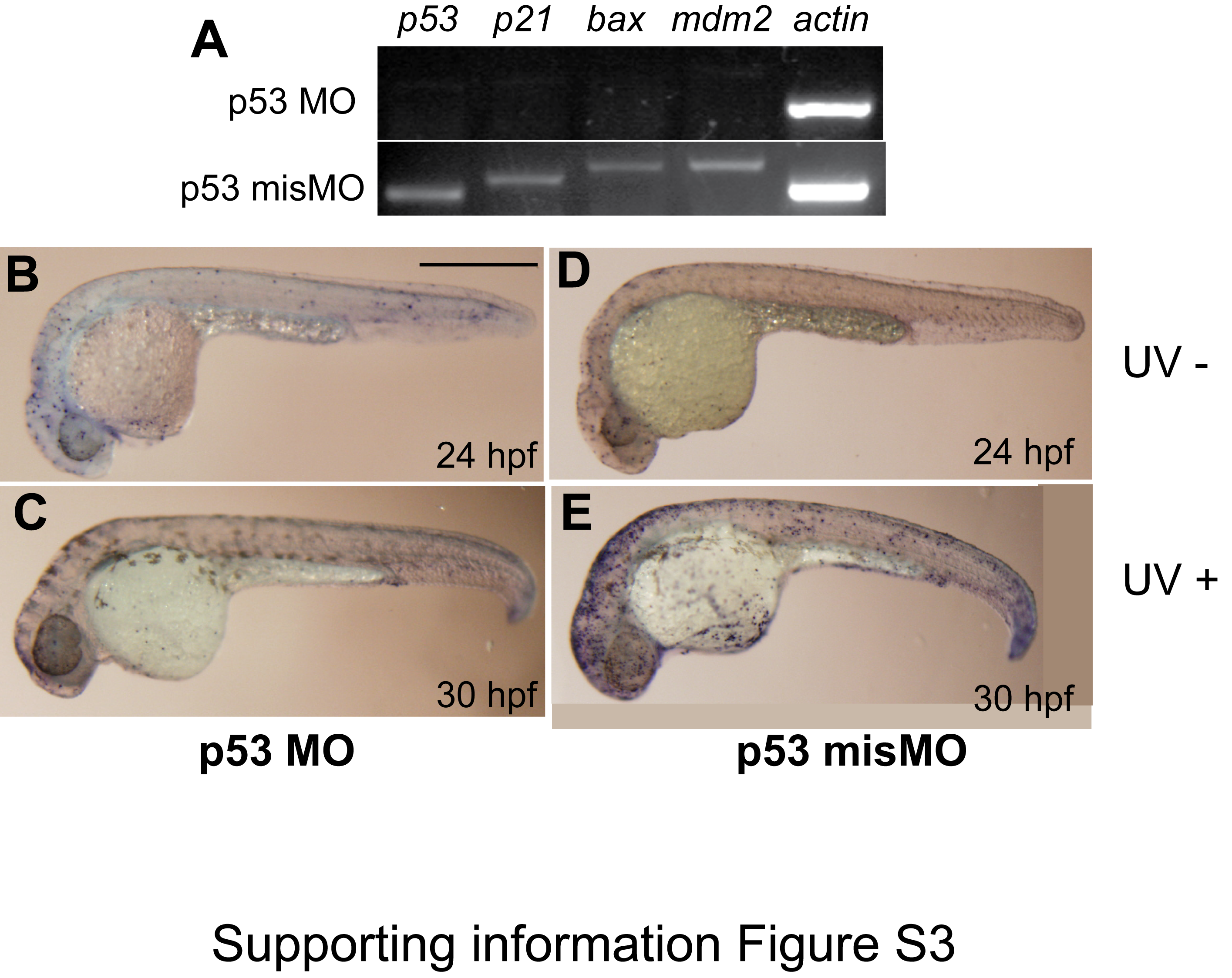

Supplement: Figure S3 — Inhibition of p53 activity and suppression of apoptosis by p53 MO. Zebrafish embryos at the one-cell stage were injected with p53 MO and p53 misMO at 50 pg/embryo. At 24 hpf, the embryos were exposed to 400 mJ/cm UV light for 5 s. Six hours later, they were fixed for TUNEL staining and RT-PCR. (A) Semiquantitative RT-PCR of p53-target genes in UV-exposed embryos injected with p53 MO (50 pg/embryo) or p53 misMO (50 pg/embryo). The p53 response genes are completely downregulated in p53 MO-injected, but not in p53 misMO-injected, embryos. (B–E) TUNEL staining of p53 MO- and p53 misMO-injected embryos before and after UV exposure. The embryos injected with p53 MO show a complete inhibition of apoptosis (indicated by an absence of TUNEL-positive cells) after UV exposure, whereas p53 misMO-injected embryos show extensive apoptosis. All images are in lateral view with anterior to the left. Scale bar: 500 µm. (3.42 MB TIF) [file pone.0004152.s005.tif]

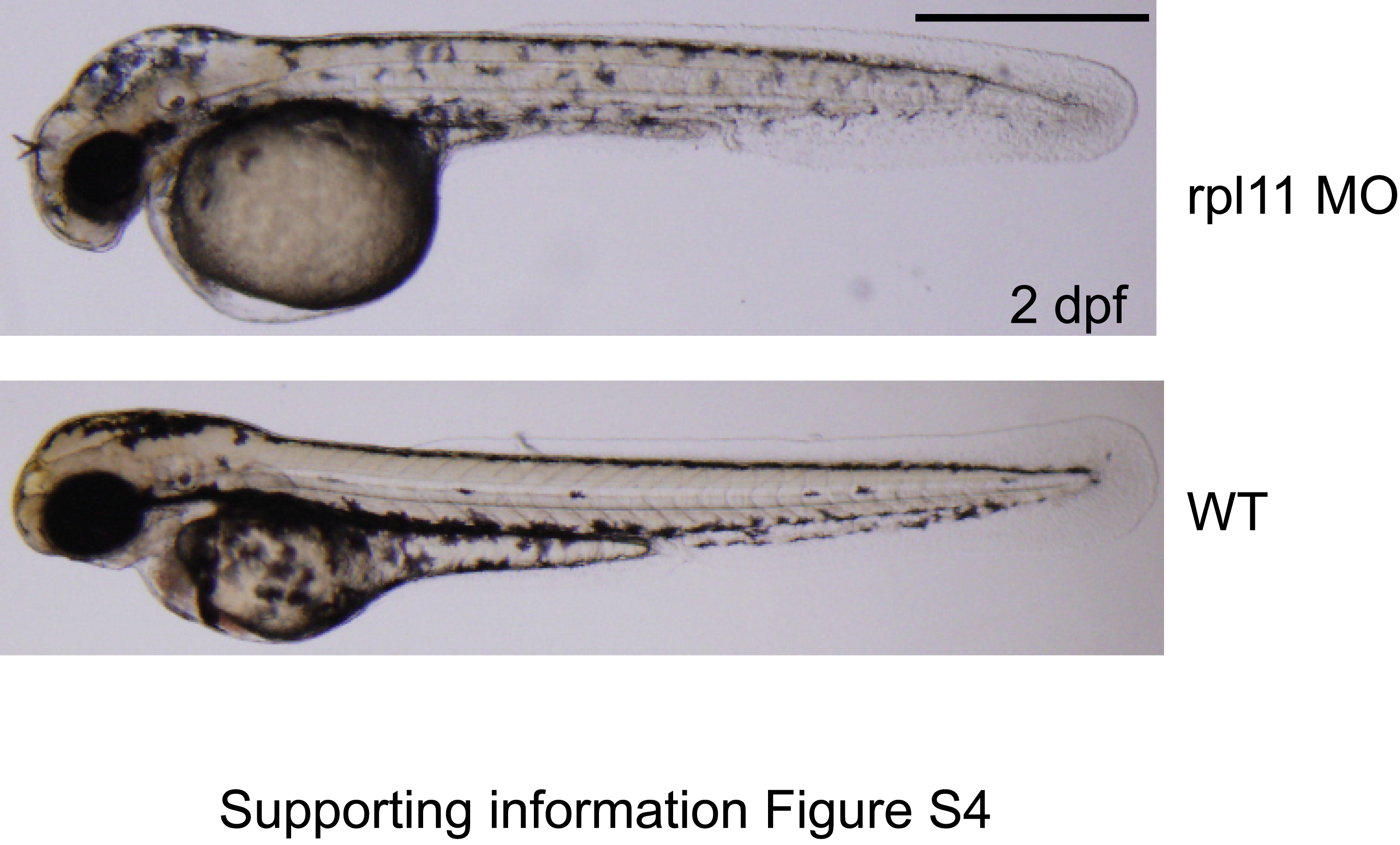

Supplement: Figure S4 — Phenotype of L11morphants at 2 day post fertilization (dpf). Morphology of L11 morphants (upper panel) shows slightly smaller head and eyes, round grey yolk with thin extension, identical to that observed in L11 mutant (rpl11hi3820btg). Lower panel is the wild-type embryo at day 2 dpf. All images are in lateral views with anterior to the left. Scale bar: 1mm (7.92 MB TIF) [file pone.0004152.s006.tif]

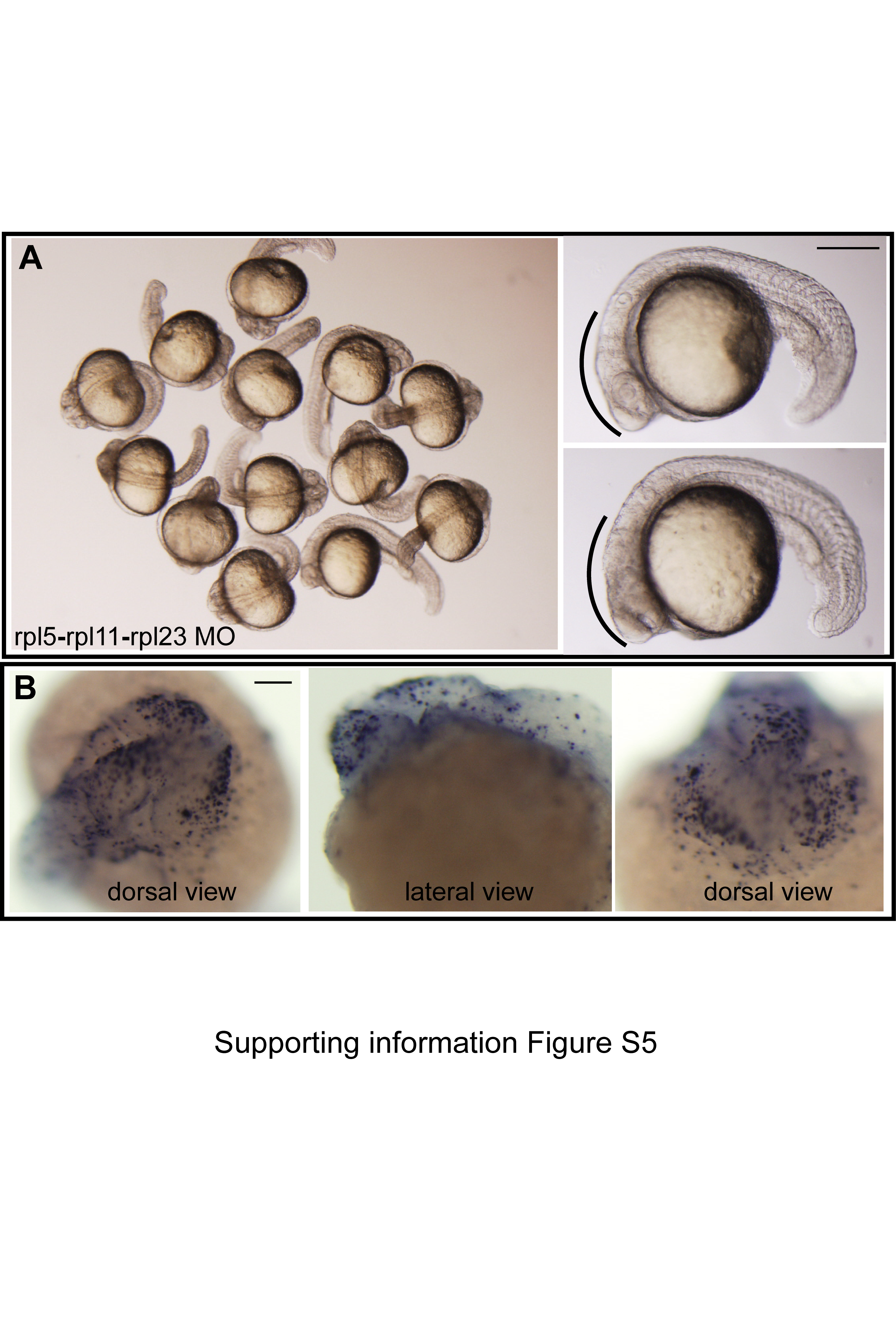

Supplement: Figure S5 — Morphological observation and TUNEL staining of embryos injected simultaneously with rpl5, rpl11, and rpl23 MOs.(A) Lateral views of 24 hpf embryos simultaneously injected with the three MOs. Severe morphological deformities with almost a complete absence of brain subdivisions (black arc) were observed in the morphants. (B) Dorsal and lateral views of the head region of 24 hpf embryos showing extensive apoptosis (blue dots). Scale bars: A, 250 µm; B, 100 µm. (8.39 MB TIF) [file pone.0004152.s007.tif]
